# Synodic lunar phases and suicide: based on 2605 suicides over 23 years, a full moon peak is apparent in premenopausal women from northern Finland

**DOI:** 10.1038/s41380-020-0768-7

**Published:** 2020-05-13

**Authors:** Victor Benno Meyer-Rochow, Tapani Hakko, Helinä Hakko, Pirkko Riipinen, Markku Timonen

**Affiliations:** 1 Department of Plant Medicals, Agricultural Science and Technology Institute, Andong National University, Andong, 36729 Republic of Korea; 2 Department of Ecology and Genetics, University of Oulu, Oulu, Finland; 3 Department of Psychiatry, Oulu University Hospital, Oulu, Finland; 4 Research Unit of Clinical 0Neuroscience, Psychiatry, Oulu, Finland; 5 Center for Life Course Health Research, University of Oulu, Oulu, Finland; 6 Unit of General Practice, Oulu University Hospital, Oulu, Finland

**Keywords:** Depression, Physiology

## Abstract

Suicide data for this study were available for the period of March 1988 to June 2011, and involved 2111 male and 494 female victims from the Finnish province of Oulu. Data for lunar phases during that period were categorised into three groups: new moon (<25% visible), full moon (>75% visible) and other times with values in between. Seasonal effects were controlled with definitions for winter (Nov, Dec, Jan), spring (Feb, Mar, Apr), summer (May, June, July), and autumn (Aug, Sep, Oct). Suicide occurrences during different lunar phases were compared with their expected distribution using multinomial tests with all tests being two-tailed. Statistical significance was set at *p* < 0.05. No correlation between suicides and moon phase in any of the four seasons was apparent for male victims, but in winter for women it was (*p* = 0.001). Further analysis of the data revealed that the full moon association was statistically significant only for premenopausal women, defined as female victims younger than 45 years of age. To explain this unexpected finding a number of factors were considered, e.g., the darkness of a northern Finnish winter with increases of SAD and depression especially in premenopausal women, the influence of the lunar periodicity on the menstrual cycle, and cosmogeophysical effects on the humoral and autonomous nervous system.

## Introduction

There can be no doubt that in animals and plants many events that are under physiological control like growth and reproduction are subject to annual, lunar and circadian rhythms [1, 2]. There is also no question that it is widely believed that the moon somehow affects the behaviour, health and emotion of people [3]. The English term “lunatic” refers to a moonstruck person who has gone crazy and somewhat similar associations between lunar phases and human reactions are found in many cultures [4, 5].

Numerous investigations have been carried out to demonstrate whether or not there really exists a relationship between lunar cycle and erratic behaviour, traffic accidents, hospital admissions, crimes and homicides, suicides and other events and the conclusion in most of these studies has been that there was no scientific evidence for any lunar effect [6–17]. In fact, their own study had led Rotton and Kelly [18] to the provocative comment that further research on the topic was unnecessary as there was no thread of evidence to support the idea of a linkage [18]. However, in spite of the denials that the moon and its phases could in any way be connected with some human activity, that view has not been shared unanimously and studies did and do exist that tend to confirm the influence of lunar phases under certain situations and conditions.

With regard to homicides in Finland it has recently been shown that contrary to most of the earlier assumptions during full moon 15% fewer homicides were committed than during the new moon phase [19]. This result held true for all seasons, weekdays, genders and ages. Contrary to a study involving 50,492 non-fatal traffic accidents in Saskatachewan that did not demonstrate a link to the lunar cycle [9], a report from Kyushu (Japan) based on 842,554 traffic accidents did show that there was indeed a significant increase in emergency transports for males aged ≥40 years of age on full moon nights before midnight [20]. An elevated risk during full moon has also been reported for motorcycle-related mortalities in the USA covering 40 years of data [21].

Evidence for a lunar influence on patients suffering from bipolar disorders has recently been presented [22, 23], which led to the speculation that links between moonlight and the reported dramatic changes in sleep duration of people with bipolar mood changes and psychoses could have led to the folklore belief of the werewolf guise [24, 25]. However, an earlier study had failed to show an influence of the lunar cycle on the amount of emergency telephone calls to a crisis centre [6], and a recent investigation involving 17,996 cases of people treated as inpatients in Switzerland also did not find statistically significant connections with either admission or discharge rates [17].

Regarding suicides the situation is no less confusing and despite the importance for the society as a whole and relatives of suicide victims in particular to know whether or not an association between the synodic lunar cycle and suicide exists, there has been very little thorough research into this question. Scrutinising 3054 suicides (1949 males and 1105 females) in Middle Franconia between 1998 and 2003 for which complete data were available, it was concluded that no relationship existed between suicides and lunar phases [11], confirming an earlier review based on 20 studies [8]. A very recent investigation poses the question “Does [the] lunar cycle influence suicide attempts?” and having examined 5647 attempted suicides in Turkey the authors also conclude that their own study and the earlier literature on the subject does not support the hypothesis of the lunar cycle having any effect on suicide attempts [26].

Moreover, yet there are reports that differ. In 1977 when all suicides in Ohio over the period of 1972–1975, tabulated by year, month, day of the week, lunar phase and holidays were analysed, a significant increase (*p* < 0.01) only with respect to the new moon phase was noted [27]. In 1973 a statistically significant lunar periodicity (*p* < 0.05) in self-inflicted injuries by poisoning in women with a greater number occurring in the first quarter and a smaller number during the third quarter of the lunar cycle had been demonstrated [28]. For males there had not been any evidence of a lunar periodicity. Differences between males and females have also been reported more recently and shown that a small but significant decrease in suicides of women during magnetic storms and full moons existed while for males over 60 years of age a significant and positive new moon effect could be identified [29]. That study was based on the daily distribution of 130,860 suicide cases (94,776 males and 36,084 females) from 1976 to 2010 in Hungary.

The discrepancies of these results are difficult to explain and could be due to differences in the methodology employed to assess the available data, to oversights like ignoring the effects of holidays, climatic events like geomagnetic storms or latitudes of the regions involved and the different years in which the investigations were carried out. Furthermore, having shown that suicide mortality in the visually impaired vis-à-vis sighted victims was significantly increased also made us wonder whether ambient luminosity and the recognition of lunar phases might not affect suicidality too, especially in a place like northern Finland with its long and dark winters [30]. This surmise was what led us to investigate whether the occurrence of suicides in the Oulu region of northern Finland was related to lunar phases and whether this phenomenon was gender-specific. Further aims were to examine (within the sample analysis) whether there existed gender differences in association with lunar phases and psychiatric disorders and whether the ages of male and female suicide victims, as well as the seasons when the suicides occurred bore any relation to the synodic lunar phases.

## Materials and methods

### Suicide data

Our suicide data cover information on all suicides (2605 suicides: 2111 men, 494 women) committed in the province of Oulu in northern Finland between March 1988 and June 2011. Basic information related to the victims was obtained from the official death certificates following obligatory medical-legal investigations. According to Finnish law such examinations are performed in every violent, unnatural, sudden or unexpected death and the forensic examiner makes the final decision to classify a death as suicide. No samples were excluded from the analysis.

The suicide data include information on date of death, age at death, gender of the victim, suicide method, history of previous suicide attempts, and alcohol intoxication at the time of death. For the purpose of this study suicide methods were classified as hanging, shooting, poisoning and other methods. Previous suicide attempts indicate at least one attempt before suicidal death. The information on being under the influence of alcohol at the time of death was based on the findings of the forensic examination.

### Care register for health care data

The basic data are linked to the data from the nationwide Finnish Care Register for Health Care (CRHC), provided by National Institute for Health and Welfare. The CRHC covers all inpatient admissions at primary and specialised level care in Finland since 1969 and contains various types of information, such as primary and subsidiary diagnoses upon discharge as well as admission and discharge dates for all treatment episodes. A register linkage is available by using the personal identity code provided to every Finnish citizen. Data for this study were accessible only to 2011 and not beyond.

The information of hospital-treated psychiatric disorders for this study was extracted from the CRHC data. A psychiatric disorder of a suicide victim was defined to be present, if at least one hospital-treatment episode due to psychiatric disorder (ICD-10: F-codes, ICD-8/9: 290-319 codes) was found in the CRHC as being a primary or subsidiary reason for admission. The Ethics Committee of the University of Oulu approved the study and the Finnish Register of the National Institute for Health and Welfare (THL) granted the release of records towards this research work.

### Lunar data

Lunar phases for each day were approximated by using the long-term average of the synodical month of 29.530588853 days. Data accuracy was confirmed by using some random samples of known full moon dates within the data set. One of the limitations of this research is that this method cannot be used for very long time series (e.g., >100 years) since the length of the synodical month constantly undergoes minor changes that are hard to predict. Calculations for each datum were carried out using MATLAB. Similar approximations have been in use since they were first introduced [31]. The data were categorised into three groups, namely: new moon if the approximated lunar phase was <25% visible, full moon when it was over 75% visible, and other times with values in between.

### Statistical analyses

The Poisson regression method was used to examine the association of lunar phases to suicides over the period of 8706 days between March 1988 and December 2011. The effect of the season was controlled in the analyses. Seasons were defined based on the amount of light intensity around solstices and equinoxes as follows: winter = November, December, January, spring = February, March, April, summer = May, June, July, autumn = August, September, October. Because of slightly different lengths of the months and a year of 365 days, it is not possible to have triplets of months that all contain exactly the same number of days.

In the within-sample analyses, statistical significance of group differences was assessed with Pearson’s Chi-square test or Fisher’s Exact test and in continuous variables with Student’s *t*-test or Mann–Whitney *U*-test. Suicide occurrences according to lunar phases were compared with expected distributions using multinomial tests (25% full moon, 25% new moon, 50% other). All tests were two-tailed and the limit for statistical significance was set at *p* < 0.05. The statistical software used in the analyses was IBM SPSS statistics version 25. Separating first and last quarters was deemed unnecessary, because the hypothesis had been about the sunlight reflected from the moon and whether lunar illumination was 50% during the first or last quarter would not have affected the brightness reaching the Earth.

## Results

### Background characteristics

A total of 2605 persons had committed suicide during the 23-year study period, being 2111 (81.0%) men and 494 (19.0%) women. Male suicide victims, in comparison with females, were characterised as younger age (mean ± SD 42.9 ± 15.9 years of age in men vs. 46.0 ± 15.9 in women, *p* < 0.001) and being more frequently under the influence of alcohol at the time of suicide (49.0% in men vs. 33.1% in women, *p* < 0.001). Female suicide victims, compared with males, had had more commonly previous suicide attempts (27.7% in women vs. 10.5% in men, *p* < 0.001) and a history of hospital-treated psychiatric disorders (59.5% in women vs. 41.6% in men, *p* < 0.001).

### Suicides by lunar phases and seasons

**Fig. 1:**
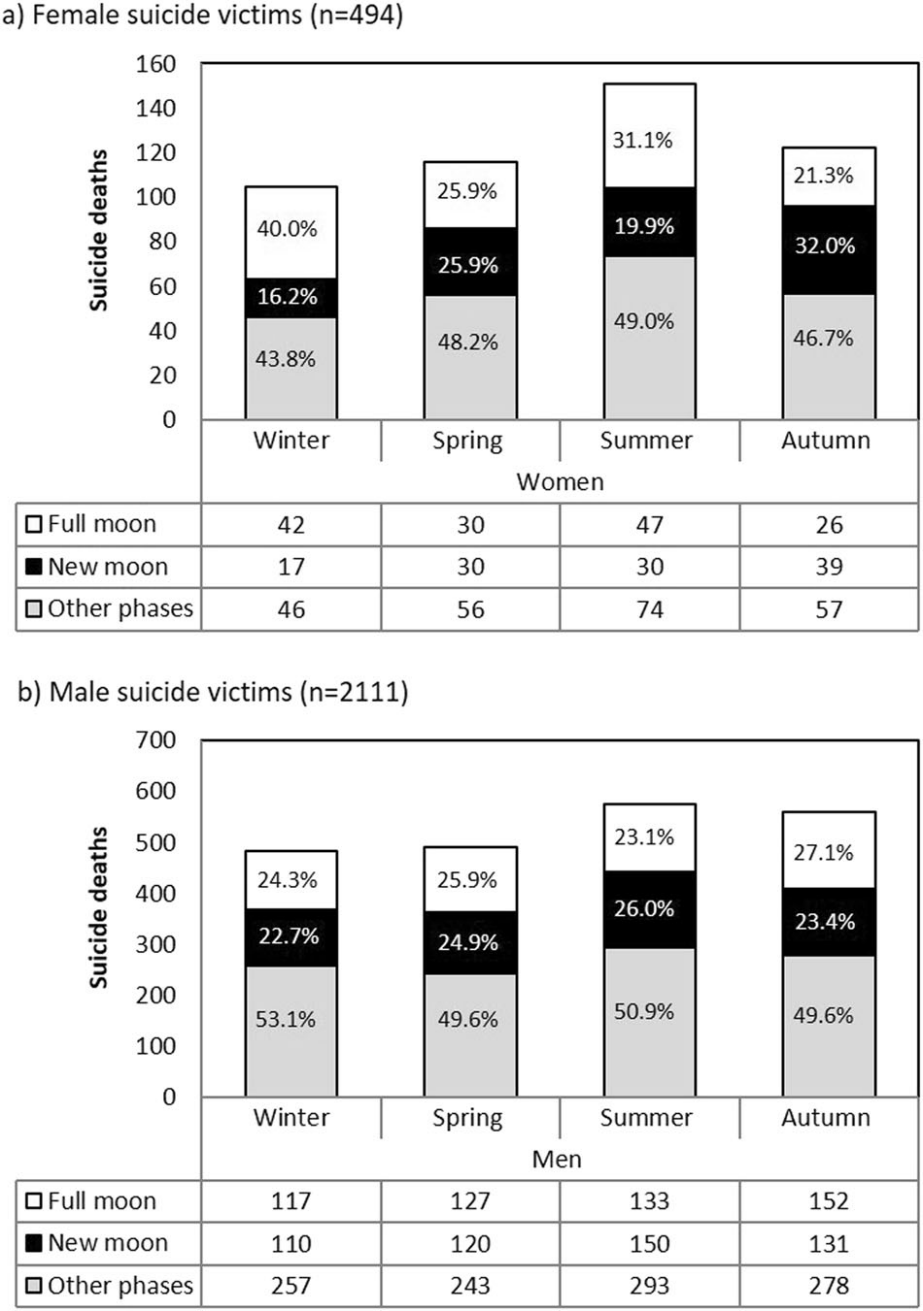
Frequency (%) distribution of suicides according to lunar phases and seasons in female and male suicide victims from northern Finland. **a** Female suicide victims (*n* = 494); **b** Male suicide victims (*n* = 2111).

Figure 1 illustrates the frequency and percentage distribution of suicides by lunar phases and seasons for male and female suicide victims. There was a statistically significant gender difference in lunar phases of suicides during winter, in which full moon accounted for 40.0% of suicides in women compared with 24.3% in men (*p* < 0.004).

**Fig. 2:**
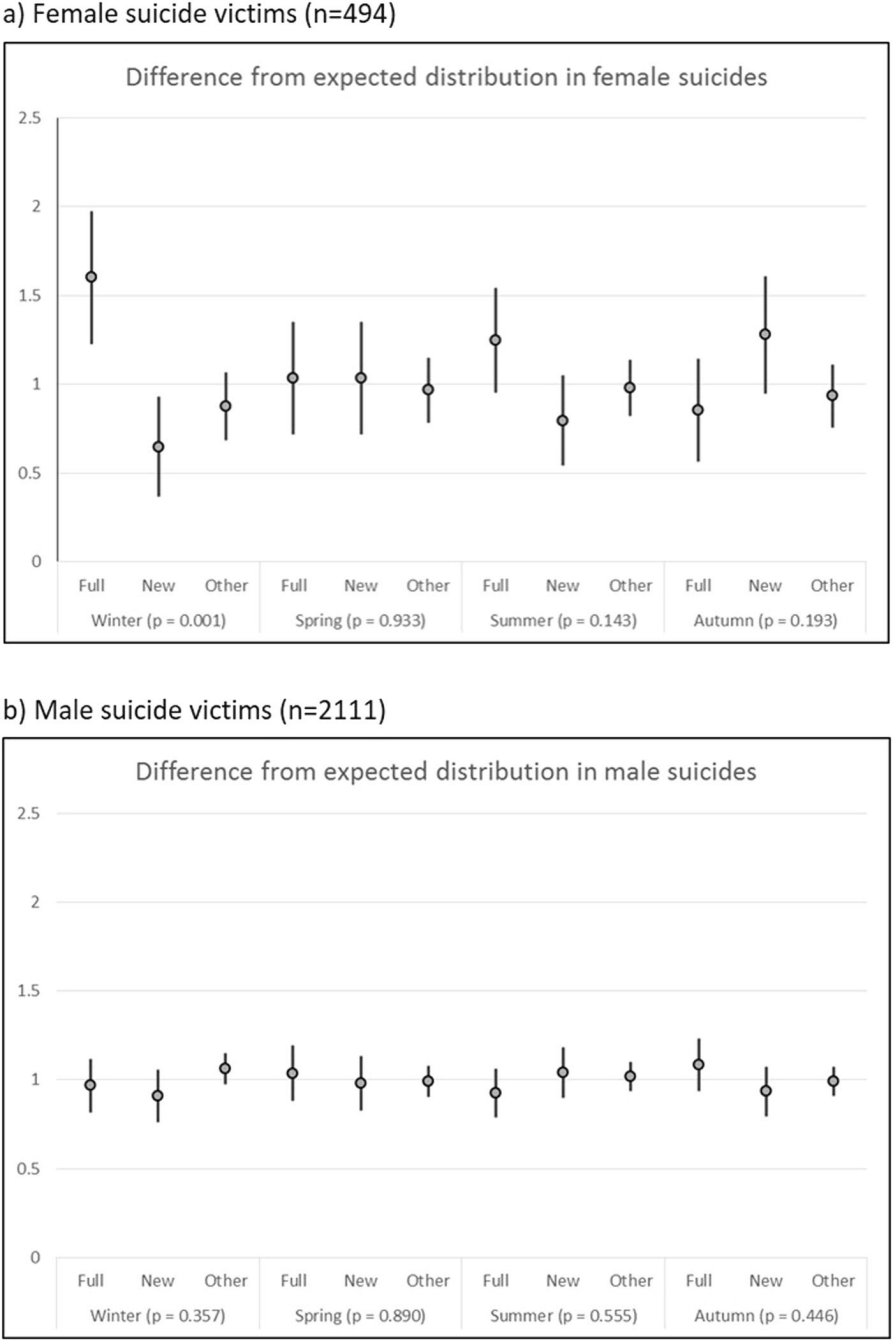
Ratios of observed to expected number of suicides according to lunar phases by season. **a** Female suicides (*n* = 494); **b** Male suicide victims (*n* = 2111).

The occurrence of suicides in relation to lunar phases was compared with their expected distribution and is illustrated in Fig. 2. In women, a statistically significant association of lunar phases with suicides during winter was observed (*p* = 0.001), showing a significant excess in winter suicides during full moon (Ratio = 1.60, 95% CI 1.23, 1.97) and a trough during new moon (Ratio = 0.65, 95% CI 0.37, 0.93). In men no association of lunar phases with suicide occurrence was found in any of the seasons.

### Poisson regression analyses for suicide counts

Table 1 shows the results of the Poisson regression analysis taking into account days without any suicide occurrences. Full moon exhibited a statistically significant correlation with suicide occurrence in women (*p* = 0.021), but not men. Thus, subsequent analyses focused solely on female suicides.

**Table 1:** Association of suicide occurrence to lunar phases among male and female victims from the northern Finland.

	Women (*N* = 494)			Men (*N* = 2111)		
Parameter	*β*	95% CI	*p*-value	*β*	95% CI	*p*-value
Lunar phase						
Ref (other lunar phases)	-	-	-	-	-	-
New moon	0.016	–0.206, 0.239	0.884	–0.041	–0.146, 0.065	0.449
Full moon	0.243	0.036, 0.450	0.021	–0.002	–0.106, 0.102	0.969
Season (covariate)						
Ref (winter)	-	-	-	-	-	-
Spring	0.101	–0.163, 0.365	0.455	0.014	–0.112, 0.139	0.829
Summer	0.334	0.085, 0.583	0.009	0.146	0.025, 0.267	0.018
Autumn	0.150	–0.111, 0.411	0.261	0.148	0.026, 0.269	0.017

Note: Poisson regression model using suicide counts as dependent variable modelled against lunar phases and seasons of the year. The model covered data of a time series of 8522 days between March 1988 and June 2011. The categorisation of seasons follows the differences in light intensity around solstices and equinoxes between seasons: Winter = Nov, Dec, Jan, Spring = Feb, Mar, Apr, Summer = May, Jun, Jul, Autumn = Aug, Sep, Oct.

Of the female suicide victims, 249 (50.4%) were of pre- and 245 (49.6%) peri/post-menopausal age. A separation of the data on female suicide victims into pre- and peri/post-menopausal women shows that a statistically significant suicide peak in relation to the winter full moon occurs only in connection with premenopausal victims (Table 2). On the other hand, a similar separation involving 1216 male victims younger and 895 older than 45 years of age showed no statistically significant differences with *p*-values of 0.578 and 0.709 for new and full moon younger victims and 0.760 and 0.312 for new and full moon older victims.

**Table 2:** Pre- and peri/post-menopausal female suicide victims.

	Premenopausal women (*N* = 249)			Peri/post-menopausal women (*N* = 245)		
Parameter	*β*	95% CI	*p*-value	*β*	95% CI	*p*-value
Lunar phase						
Ref (other lunar phases)	-	-	-	-	-	-
New moon	0.017	–0.299, 0.333	0.916	0.016	–0.297, 0.329	0.920
Full moon	0.298	0.009, 0.587	0.043	0.185	–0.111, 0.471	0.221
Season (covariate)						
Ref (winter)	-	-	-	-	-	-
Spring	–0.128	–0.504, 0.248	0.506	0.325	–0.050, 0.701	0.089
Summer	0.267	–0.073, 0.607	0.124	0.411	0.045, 0.778	0.028
Autumn	0.066	–0.292, 0.424	0.717	0.244	–0.138, 0.626	0.211

Note: Poisson regression model using suicide counts as dependent variable against lunar phases and seasons of the year. The model covered data of a time series of 8522 days between March 1988 and June 2011. The categorisation of the seasons follows the differences in light intensity around solstices and equinoxes between seasons: Winter = Nov, Dec, Jan, Spring = Feb, Mar, Apr, Summer = May, Jun, Jul, Autumn = Aug, Sep, Oct. Women are grouped according to age criteria; premenopausal women were <45 years old and peri/post-menopausal were 45 years of age or older.

A history of hospital-treated psychiatric disorder was recorded for 294 (59.5%) of the female suicide victims. A marginally significant association of full moon with suicide occurrence was noted for women with a history of hospital-treated psychiatric disorders (*p* = 0.078), but the percentages of patients having received treatment for psychiatric disorders was statistically not significantly different between pre- and peri/post-menopausal women.

## Discussion

There are no surprises in our data related to the different methods used by male and female suicide victims and the fact that ~4 times more males than females committed suicides; observations such as these had been reported earlier [32]. However, our finding of a male/female difference from the expected distribution of suicides throughout the year and the finding that only females show a significant winter increase of suicides during full moon (and a minor decrease at new moon) need to be addressed.

The inhabitants of our study region, the province of Oulu, experience summers with very short or almost no nights but opposite conditions in winter. The photic environment is therefore one factor that has to be considered. The prolonged winter darkness affects people in various ways and the Finnish term “kaamos” is a word with huge emotional power. In Finland “kaamos depression” is recognised as a seasonal affective disorder (SAD) of the winter type and according to Sandman et al. women “generally experience higher prevalence than men” [33].

Although studies based on cases in Norway and the USA found a positive correlation between SAD and latitude [34, 35], others did not [36, 37]. However, it was shown that a higher risk to develop seasonal symptoms existed for woman and that this risk decreased with aging in Sweden [38]. It was pointed out that “later sunrise times relative to sleep times are triggering depressive episodes” and depressive episodes have long been identified as a major suicidal risk factor [39]. It has been argued that the delayed or missing “dawn signal” is critically involved in triggering SADs and that an artificial dawn signal presented to patients during the final hours of sleep on long winter nights worked as an antidepressant [40].

On the basis of these earlier reports, we can accept that women are more likely than males to be affected by the seasonal winter depression and consequently at that time may be at a greater risk of committing suicide than men. However, the question remains why only females, and more precisely premenopausal females, exhibit our winter suicide peak at full moon. The fact that SAD and premenstrual depression (PMDD), beginning with luteal onset, have common symptoms make it likely that those suffering from both disorders are at a higher suicide risk during wintertime [41].

The close similarity of the synodic lunar with the menstrual cycle comes to mind and although, as with the lunar effect on suicides, contradictory results have been published in connection with lunar and menstrual periodicities, the lengths of the two cycles are too similar to be ignored. Yet, the few studies in which female suicides, but not those of males, were shown to exhibit some relationship to the phase of the moon did not show any coherent trend: a statistically significant lunar periodicity of self-inflicted poisoning in Winnipeg (Canada) has been reported to be greatest for women during the first quarter of the lunar cycle [28], while Kmetty et al. [19] reported fewer suicides on the day of full moon than could be expected, but only for post-menopausal females aged 50–59 years. The latter result agrees with our suicide finding (this paper) and also some earlier reports that older, post-menopausal women were less affected by season-bound depressive symptoms [38, 42].

The prolonged darkness in the northern Finnish winter necessitates longer exposures to artificial light sources and a lack or delay of the dawn light. The unnatural photic environment with a lack of the UV-component will not only affect melatonin secretion and the effectiveness of this hormone (people tend to sleep more and longer, but not better and abnormally long sleep phases are typical for depression) [25], but other physiological regulators as well. Schwartz et al. [43], for example, suggested that seasonal moods and behavioural changes were related to changes in serotonergic and hypothalamic-pituitary adrenal functions, and it has also been reported that suicidal attempts were most frequent among females during the menstrual phase [44, 45]. Suicide attempts in premenopausal women were most common when oestrogen levels were lowest, i.e., during late luteal and follicular phases [46]. If, as asserted [47], ovulation occurs predominantly in the dark phase of the lunar period or under the decreasing illumination after full moon [48], then 14 or 15 days later we would be at the beginning of a new menstrual cycle precisely at full moon. There is, however, no consensus as to whether there is any synchrony between the menstrual cycle and the moon phase and the existence of such relations are not universally accepted [49, 50].

Of the three consequences that the lunar cycle has on the Earth’s human inhabitants, the moon’s luminance per se is unlikely to be the sole reason for the increase in premenopausal female suicides during full mean in northern Finland or any concomitant psychiatric condition. Gravitational cycles, however small, may be perceived by the human vestibular system [51], and effects from the moon’s wake on the magnetosphere have been implicated in connection with a variety of lunar biological effects [52]. Geomagnetic storms can trigger strokes [53], and a significantly higher rate of suicides by females but not males during times of geomagnetic storms has been reported [54], in contrast to a finding of a decrease (but in a considerably narrower age-group) [29]. For a study on the cosmogeophysical effects in the Arctic, unfortunately involving only male subjects (12 men aged 19–38), the conclusion was that despite wide individual differences emotional states were clearly subject to central and autonomous nervous system and humoral effects “modulated…by the cosmogeophysical and meteorological agents” [55].

Pre- and post-menopausal women differ with regard to the menstrual period, which is “a carefully orchestrated sequence of interactions among the hypothalamus, pituitary, ovary, and endometrium” [56]. That the moon in connection with cosmogeophysical factors like lunar phases and geomagnetic storms can affect calcium flux, melatonin, steroids, etc. is considered likely [52, 57]. Although our study cannot confirm that such lunar effects were indeed the reason for the winter full moon suicide peak in premenopausal victims, we cannot dismiss this possibility. However, we do suggest that other studies into the possible link between suicides and lunar phases not only analyse their data according to gender, but also separate victims into pre- and post-menopausal women, check for seasonal variations and obtain a record of geomagnetic activities during the date of a suicide. We should remember that rather than ignoring folklore, the latter can encourage scientists to probe and then verify or falsify what “common sense” has been claiming for centuries [3].
